# HIV-Related Sexual Behaviors among Migrants and Non-migrants in Rural Ethiopia: Role of Rural to Urban Migration in HIV Transmission

**Published:** 2011-12

**Authors:** Melesse Tamiru, Damen Hailemariam, Getnet Mitike, Jemal Haidar

**Affiliations:** *School of Public Health, College of Health Sciences, Addis Ababa University, Ethiopia*

**Keywords:** bridging, Ethiopia, HIV risks, migrants, non-migrants, sexual risk behavior

## Abstract

**Objective:**

To compare HIV-related sexual risk behavior among temporary rural to urban migrants and non-migrants and to explore the role of migration in HIV transmission in a rural area of Ethiopia.

**Methods:**

A cross-sectional comparative study was conducted in Bure Woreda, West Gojam, Amhara Region, Ethiopia. A total of 1,310 male subjects (655 rural to urban migrants and 655 non-migrants) were selected randomly and were assessed, analyzed using SPSS version 17 software for their HIV related sexual risk behaviours including the role of migration in HIV transmission in a rural Ethiopia. Two parts of questionnaires were prepared and used for comparing the above groups. The first part of the questionnaires included non-sensitive questions such as demographics and HIV knowledge while the second part comprised sensitive questions related to sexual behaviors.

**Results:**

When multiple sexual partners, sex with commercial sex workers, sexual transmitted infections and premarital sex compared between the two groups, the proportions of rural to urban migrants Vs non- migrants who had multiple sexual partners (31.4 % Vs 7.4 %), sex with commercial sex workers (22.3% Vs 13.3%), sexual transmitted infections (11.7% Vs 3.2%) and premarital sex (20.8% Vs 14.2 %) were significantly higher in rural to urban migrants than non-migrants. Among those who had multiple sexual partners, only 12.7 % of, rural to urban migrants and 9.8 % of non-migrants reported consistent condom use with sexual partners other than their spouse.

**Conclusions:**

As both rural to urban migrants and non-migrants are at risk for HIV infection, intervention programmes targeting both groups are recommended. However, in order to contain the bridging effect on HIV transmission from urban to rural areas particular attention should be given for the rural to urban migrant population.

## INTRODUCTION

Globally around 33.3 million people are living with HIV and Sub-Saharan Africa remains the region most heavily affected by HIV, accounted for 68% HIV infections and 72% AIDS-related deaths in the world (1).

In Ethiopia based on the 2007 single point HIV prevalence estimates, the national HIV prevalence for 2008 is estimated 2.2%. It is also indicated that the national and rural HIV prevalence of the country has stabilized while the urban prevalence is declining. Although 84% of the population lives in rural areas the majority of studies on HIV/AIDS are done in urban settings, and only few studies are available for the rural population of Ethiopia. The Amhara region is reported as one of the most HIV stricken in the country. The urban and rural HIV prevalence for the year 2005 for the region were estimated at 13.5% and 3.2% respectively (2). And the rural residents in the region had less knowledge about the disease than urban residents (2). It has been reported that the proportion of condom use is low and the vast majority of farmers perceived themselves to be at low risk of HIV infection (3). The prevalence of HIV among most at risk populations (MARPS) including migrants in hot spot areas in the region is ranging from 11.6% to 37%, which is five- fold higher than the national urban adult population (7.7%) (4). The high prevalence is attributed to sexual partner change, multiple sexual partnership, high exposure to STIs, and low and inconsistent condom use (5).

According to the 2005 Ethiopian Demographic Health Survey (EDHS), the small towns including Bure town was identified as HIV hot spot and exhibited higher prevalence of HIV as compared to the bigger towns in Ethiopia. The proximity and surrounding of this small town (Bure) by the adjacent rural kebeles could pose a greater risk to the unaffected rural populations (6). The people from the study area (rural kebeles) have frequent contact with the town for temporary jobs (7) which is creating an opportunity to explore mechanisms of urban to rural spread of HIV risk. This type of seasonal migration is most often undertaken by farmers during the slack season and return home during agricultural activities and this population is described as ‘bridge populations’ for the HIV epidemic in rural areas (8) and considered as one of the various social factors that have contributed to the HIV epidemic (9-11).

Several studies have shown that people who are more mobile or who have recently changed residence tend to be at higher risk for HIV and other sexually transmitted infections (STIs) than people in more stable living arrangements (12-15).

The relationship between population migration and the spread of HIV is well described (16, 17). However, to date, the reasons for this relationship have not been well explored. Some researchers have found no difference in sexual behaviors between migrants and non-migrants (18), while other studies have documented a correlation between the migration experience and increased sexual risk behaviors (19-22) and speculated on a number of social and cultural conditions that could potentially contribute to the link between rural to urban migration and sexual risk (19-23). Still, the precise way in which migration contributes to the spread of STIs and HIV infection is complex and not well understood. Previous studies have focused on the destinations of migrants, or, less often, on the areas from which migrants come (24). Few other studies have considered both ends of the migration process of those who leave home as well as those who remain behind. Understanding the need to explore both ends of migration routes as crucial steps for successful interventions. Therefore this study has addressed the above identified gap in the country by comparing the HIV related sexual risk behaviors of rural to urban migrants and non- migrants including the role of migration in the spread of HIV risk to the rural community.

## METHODS

### Description of the Study Area

The study was conducted in Amhara region which is the second largest and second most densely populated region in the country located in the northern part of Ethiopia. The region is administratively divided in to 11 administrative zones and 133 districts (woredas) and about 3,232 villages known as kebeles. Bure is amongst the woredas in the region which is included in this study for its high HIV prevalence and the presence of most at risk population in the Amhara region (4). It is located 148 km southwest of the capital of the region Bahir Dar and 400 km northwest of the capital of the country, Addis Ababa. The woreda has a total of 23 rural kebeles with an estimated population of 281,310 (141,683 males & 139,627 females). It is along the main route to other major towns and is frequented by businessmen from the HIV hot spot areas. The town hosts several population groups such as farmers, rural migrant labourers, local businessmen, and youth having sex with female sex workers at increased risk of contracting HIV and a long-established tradition of married men having informal parallel families (Kemete) in the town (4).

### Design

A cross-sectional, community based comparative study conducted from November, 2010 to January, 2011.

### Sample Size

The required sample size was determined using STAT-CALC program of the EPI INFO statistical package with 5% type I error and 90 % power and one to one allocation ratio of rural to urban migrants to non-migrant group (n1: n2) were assumed. With prevalence of risk behavior among non-migrants of 30 % taken from EDHS, 2005 (2) and prevalence of risk behavior among rural to urban migrants 39 % [A difference of 9 percent was assumed to exist for lack of similar studies] and non response rate of 10%. A total of 1,333 study subjects (666 rural to urban migrants and 667 non- migrants) were estimated.

### Sampling Procedures

Simple random sampling was applied to select the two groups of study participants from the prepared fresh list of the sampling frame. For non-migrants (rural residents) the total sample was equally selected from the 23 rural kebles of Bure woreda and from each kebele 29 respondents were selected randomly from the sampling frame which was prepared from the census. Likewise for rural to urban migrants the total sample 666 was selected from four working groups (167 road construction (cobble stone) rural to urban migrant workers, 166 Ethiopian Commodity Exchange (ECX) rural to urban migrant workers, 167 Birshelko rural to urban migrant farm workers and the rest 166 were leg Migra rural to urban migrant farm workers).

### Study Tools and Data Collection Procedures

A pre-tested questionnaire adopted from behavioral studies on HIV/STIs was used to assess the knowledge, perception and sexual practice of rural to urban migrants and non-migrants towards HIV/AIDS (3). The questionnaire was prepared initially in English and translated to Amharic (the Ethiopian national language). Twenty three interviewers who had completed diploma level of education were recruited and trained intensively for seven days in the study area. Data were collected from both groups of the participants at the rural household level for non-migrants and at the working sites for rural to urban migrants in Bure town and its outskirt under close supervision of the principal investigator and one field coordinator.

The working sites served as the sampling unit, and owners or managers of the organizations were contacted for permission to conduct the study at their premises. Trained outreach interviewers approached individual migrant worker and invited them to participate in the survey after explaining the purposes, procedure, potential benefits and risks of the study. To get maximum responses data were collected in private places through face-to-face interviews.

### Study Participants

Both rural to urban migrants and non-migrants were enrolled. Since females were very few and the vast majority of migrants were males only males were enrolled. The male migrants temporarily working in the study area were recruited from road construction sites (cobblestone), ECX and private agricultural farms based on the following eligibility criteria:

- Male temporary rural to urban migrants who have families in rural kebeles of Bure Woreda and without a permanent town residence at the time of the interview.
- Having identity card from one of the 23 rural kebeles of Bure woreda and who were working more than one month at the study sites (Cobblestone, ECX and private agricultural farms);
- Aged 18-49 years; While the non-migrant participants were drawn from the local residents (rural kebeles) based on the following eligibility criteria;
- Males who had permanent rural residency;
- Primarily engaged in agriculture or farming-related activities;
- Aged between 18 and 49 years and did not have a history of migration (return migrants).

When there were multiple eligible individuals within a single household who met the above selection criteria, the first person who was contacted by the interviewer and agreed to participate was recruited.

### Data Analysis

Data were entered and cleaned using Epi-info version 6 and transported to SPSS version 17 for descriptive and inferential analyses. Statistics is presented in percentages. Chi-square test was employed to examine differences of socio-demographic factors between rural to urban migrants and non-migrants (e.g. age, gender, education and marital status).

To ascertain the association between the dependant variable sexual risk behaviors and the explanatory variables of both groups, simultaneously controlling for age, gender, education, marital status, and family socioeconomic status, logistic regression was run and results are presented using crude odds ratio (COR), Adjusted odds ratio (AOR) and confidence intervals (95% CI). In all analyses, *P*<0.05 was considered significant.

### Ethical Clearance

This study was approved by the Institutional Review Board of the Faculty of Medicine, Addis Ababa University, Ethiopia. A written informed consent was obtained by thumbprint or signature from each study participant. After obtaining informed written consent and each session lasted approximately 30 minutes. The interviews were conducted anonymously in an isolated room or a quiet place in order to minimize embarrassment related to discussion of sensitive topics.

### Operational definitions/measurements

- **Temporary (seasonal) rural to urban male migrants:** Those road construction site workers/farm workers who work far away from their permanent places of residence (rural area) and are usually unable to return home at the end of the work day. They, therefore, have temporary residences in the vicinity of their work sites (town) and return home at various intervals.
- **Bridging Populations:** Temporary rural to urban migrant adults (men) who link their rural communities to higher-risk urban (town) hinterlands for temporary employment. They are seasonal migrants who seek alternative employment during the quiet months in farming, and engage in road construction sites or in commercial agricultural farms around Bure town and usually remain at destination from one to six months.
- **Non-migrants:** Rural residents’ were asked whether they had a history of migration. Those who did not have a history of migration were coded as non-migrants.
- **Perceived vulnerability to HIV infection** was measured by the following question: 1) ‘People have different ideas about their risk of getting HIV/AIDS. What do you think the chances are that you will acquire HIV?’ Response choices were: 1) No chance, 2) Low chance, 3) Moderate, 4) High chance.
- **Comprehensive HIV knowledge:** this index was built based on the answers to the following six questions: three questions on Knowledge of Prevention and three questions on common misconceptions (beliefs). A Person was considered as having comprehensive knowledge if he knew all the three preventive methods and rejected the three misconceptions.
- **Knowledge of HIV Prevention Methods:** Knowledge of Prevention included three questions measuring (abstinence, being faithful and condom use):
  Can people protect themselves from HIV, the virus that causes AIDS by using condom correctly every time they have sex?
  Can People protect themselves from HIV, by having one uninfected faithful sexual partner? (excluding other transmission routes).
  Can People Protect themselves from HIV having one uninfected faithful sexual partner? (excluding other transmission routes).
  **Misconceptions to three:** Knowledge on misconceptions was measured if a person had rejected three common misconceptions. These misconceptions were common in the country.
  Can a person get the HIV virus from mosquito bites?
  Can a person get the HIV virus from eating raw meat prepared by a person infected with HIV?
  Do you think that a healthy-looking person can be infected with HIV, the virus that causes AIDS?
- **Sexual risk behaviors** were assessed by questions regarding: 1) premarital sex (yes/no), 2) multiple sexual partners (yes/no; defined as having had more than one sexual partner), 3) consistent condom use with multiple sexual partners (yes/no) and having sexual intercourse with commercial sex workers (yes/no).
- **Kemete (parallel families):** Economically well-to-do married men establish long-term sexual relationships with young women whom they support financially.
- **Consistent condom use** – use of a condom during every sexual encounter.

## RESULTS

Altogether a total of 1,333 participants (666 rural to urban migrants and 667 non-migrants) were invited to participate in the study, of whom 23 (1.7 %, 23/1, 333) were not available as the time of the survey despite repeated revisits. Their non-availability however was not related to this particular study. Thus, 1,310 participants (655 each from rural to urban migrants and non-migrants) were interviewed. Table 1 presents the socio-demographic characteristics of the rural to urban migrants and non-migrants. As shown, more than half (58.2%) of the migrants were young (18-27 years), had no formal education (48.7%) and most (55.3%) were wage laborers. Over half of migrants were married (54.4), and the rest were single (29.6 %) and divorced (16.0%). The proportion of migrants who had parallel families (kemete) were, (5.0%) and HIV knowledge 22.9 % was higher in migrants than non-migrant (4.0% and 9.5 respectively) but the difference noted was not significant (*p*=0.2, 0.13). Compared with the non-migrants, more migrants were younger (58.2%), completed secondary schooling (16.3%) and were wage labourers (55.3%) than the non-migrant (34.4%, 8.5% and 0% respectively) and the differences noted were statistically significant in all the variable (*P*=0.001).

**Table 1:** Socio-demographic characteristics of rural to urban migrants and non-migrants in Bure Woreda, West Gojam, Amhara Region, Ethiopia, 2011

Variables	Rural to urban migrants		Non-migrants		Chi-square value	*P* Value
	N	%	N	%		

Age						
18-27	381	58.2	156	23.8	172.69	0.001
28-37	162	24.7	225	34.4		
38-47	93	14.2	222	33.9		
>47	19	19.0	52	7.9		
Education						
No education	319	48.7	358	54.7	18.68	0.001
Read and write	30	4.6	33	5.0		
Primary School	199	30.4	208	31.8		
Secondary school and above	107	16.3	56	8.5		
Marital Status						
Married	356	54.4	591	90.2	244.86	0 .001
Divorced	105	16.3	36	5.5		
Widowed	-	-	9	1.4		
Single	194	29.6	19	2.9		
Monthly household expenditure in Ethiopian Birr						
≤300	225	34.4	410	62.6	0.27	0.001
301-600	415	63.4	190	29.0		
601-900	7	1.1	18	2.7		
≥901	8	1.2	37	5.6		0.001
Comprehensive knowledge of HIV/AIDS						
Yes	150	22.9	62	9.5	43.58	
No	505	77.1	593	90.5		
Having Parallel Families						
Yes	34	5	27	4.0	0.84	0.21
No	621	95	628	96.0		
Occupation						
Agriculture	258	39.4				
Student	21	3.2	645	98.5	545.7	0.001
Service provider	14	2.1	10	1.5		
Wage labourer	362	55.3				

The HIV/AIDS risk behaviours among rural to urban migrants and non-migrants are depicted in Table 2. The proportions of migrants who had premarital sex (20.8%) and multiple sexual partners (MSP) (31.40%) were significantly higher than those of non-migrants (7.4% and 14.2% respectively). Among those who had multiple sexual partners, only 12.7% of migrants and 9.8% of non-migrants reported consistent condom use with their sexual partners.

**Table 2:** HIV/AIDS sexual risk behaviors among rural to urban migrants and non-migrants in Bure Woreda, West Gojam, Amhara Region, Ethiopia, 2011

Variables	Rural to urban migrants		Non-migrants		Crude odds ratio (COR) (95%CL)	Adjusted odds ratio (AOR) (95%CL)
	N	%	N	%		

Multiple sexual partners (MSP)						
Yes	118	31.4	41	7.4	3.74(2.26-4.78)^a^	4.89 (3.13-7.66)^a^
No	258	68.6	515	92.6		
Consistent Condom Use with MSP						
Yes	15	12.7	4	9.8	1.34(0.42-4.31)	1.32 (0.28-6.08)
No	103	87.3	37	90.2		
Sex with commercial sex workers						
Yes	84	22.3	74	13.3	1.87 (1.32-2.64)^a^	0.84(0.50-1.42)
No	292	77.7	482	86.7		
Sexual transmitted infections						
Yes	76	11.7	18	3.2	3.95 (2.25-6.96)^a^	5.03 (2.72-9.30)^a^
No	300	88.3	538	96.8		
Premarital Sex						
Yes	136	20.8	93	14.2	1.58 (1.18-2.11)^a^	0.99 (0.34-2.86)
No	519	79.2	562	85.8		

a Statistically significant at *P*<0.05.

Sexual transmitted infection was higher in migrants (11.7%) than non migrants (3.2%). In the crude analysis except condom use MSP, STIs and premarital sex were significantly higher in the migrants than the non-migrants; nevertheless, when adjusted for other confounding effects, only MSP and STIs remained significant. The likelihood of having multiple sexual partners was 4.89 times higher in migrants than non-migrants (AOR=4.8; 95%CI=3.13-7.66). Similarly, the likelihood of having sexual transmitted infection was 5.03 times higher in migrants than non-migrants (AOR=5.03; 95%CI=2.72-9.30).

Table 3 depicts results from multiple logistic regression analysis on sexual risk behaviors among migrants adjusted for other factors (age, education, marital status, months spent in migratory town and frequency of migration and comprehensive knowledge), having multiple sexual partners (MSP) was significantly associated with being single, agricultural job and wage laborer.

**Table 3:** Factors associated with having multiple sexual partners, among rural to urban migrants in Bure Woreda, West Gojam, Amhara Region, Ethiopia, 2011

Variables	Multiple sexual partners			Variables	Multiple sexual partners		
	N	%	AOR (95%CI)		N	%	AOR (95%CI)

Age				Occupation			
18-27	381	58.2	0.53 (0.10-2.78)	Agriculture	258	39.4	3.26 (1.79-5.93)
28-37	162	24.7	0.67(0.13-3.45)	Student	21	3.2	0.26 (0.37-1.83)
38-47	93	14.2	0.74 (0.13-4.22)	Service provider	14	2.1	0.19 (0.02-1.44)
>47	19	2.9	1	Wage labourer	362	55.3	1
Education				Frequency of migratory town visits			
No education	319	48.7	0.46 (0.12-2.71)	1 time	107	16.3	1.08 (0.47-2.51)
Read and write	30	4.6	0.25 (0.42-1.51)	2 times	149	22.7	1.37 (0.65-2.88)
Primary school	199	30.4	0.50(0.14-1.83)	3 times	153	23.4	2.01 (0.98-4.10)
Secondary school and above	107	16.3	1	>4 times	246	37.6	1
Marital Status				Perceived risk for HIV infection			
Married	356	54.4	1.8(0.84-3.85)	No chance	462	70.5	6.04 (3.74-10.84)
Divorced	105	16.0	0.35 (0.13-0.92)	Low chance	101	15.4	0.82(0.26-2.61)
Single	194	29.6	1	Do not know	68	10.4	0.44 (0.39-8.67)
				High chance	24	3.7	1

The divorced were 65% less likely to report having had multiple sexual partners than singles (AOR=0.35; 95%CI=0.13-0.92). Similarly, the likelihood of having MSP was 3.26 time higher in migrants who had agricultural job than being wage labourer (AOR=3.26; 95%CI=1.79-5.93). Furthermore the likelihood of having MSP was 6.04 times higher in migrants who responded that they had no chance of getting HIV than those who responded high chance (AOR 6.04, 95%CI=3.74-10.84).

## DISCUSSION

This study is among the first to directly compare HIV-related sexual risk behaviors and perceptions among temporary rural to urban migrants and non-migrants in Ethiopia. Both temporary ruralto urban migrants and non-migrants appeared to be at risk of HIV infection though the magnitude is higher among the rural to urban migrants. This is in concordance with the Ugandan findings. According to the Ugandan study, people who had moved within the last five years were three times more likely to be infected with HIV than those who had lived in the same place for more than 10 years (25) and in South Africa, people who had recently changed their residence were 3 times more likely to be infected with HIV than those who had not (25). Decosas and others have argued in that it is not solely the movement itself but rather the conditions and structure of the migration process that put people at risk for HIV and other STIs (9, 26).

The role of migration in the spread of HIV has been described primarily as a result of men becoming infected while they are away from home and infecting their wives or regular partners when they return. This assertion was documented by Pison in his seasonal migration study conducted in Senegal (27). Similar findings are also found in this study where frequent back and forth of migration was seen and this is an important consideration for future program initiatives. The fact that migrant population is growing in Ethiopia, this study depicts that migrants play the potential role in spreading HIV between rural and urban areas as the migrants move periodically from their rural residence to urban cities in search of jobs.

Most young migrants originate from rural villages, where their social and sexual behaviors are strongly influenced and controlled by their families and culture. After migration, they are no longer under the control of their families and community. Our data showed a substantial proportion of migrants were in under these characters. Furthermore they had no education and were single indicating that the migrants could be potential bridging population /population at risk to transmit HIV. Some studies have shown that temporary rural to urban migrants in China who are at risk of HIV are predominantly single males in their late teens through early 30s (28-30). Suggesting the need for appropriate education targeting the group.

As males are generally more prone to risk-taking than females and young and single adults are more adventurous than older and married people, the sex, age and marital status selectivity of temporary migrants would suggest that they are more likely to have risky behaviors than non-migrants. For instance, migration in China is described as ‘circulation’ (31). Migrants travel frequently back and forth between their migration destinations and their hometowns. While four-fifths of HIV cases are reported in rural areas in China, the majority of cases of STIs have been reported in urban areas (32). In the same study it was reported that the frequency of hometown visits was significantly associated with the acquisition of STIs among temporary rural to urban migrants.

Because temporary rural to urban migrants are observed to serve as a bridge for the HIV/STI epidemics between urban and rural areas, a concerted effort is required that limits the migration through creation of job opportunities in their residential area. The role of migration in the spread of HIV to families in rural areas has been reported in other developing countries which are concordant with the present study findings (33, 34). Although the acquisition of STIs among migrants was significantly higher than their counterparts, even small proportion of STI is unacceptable in non-migrants because of its negative health consequences and its association with HIV/AIDS. Moreover, some of the non-migrants reported having premarital sex; exposed to multiple sexual partners; and the low level of condom use are alarming for HIV risk. Again highlights the need for the awareness creation, promotion of condom use and advice on safer sex. It is apparent that, migrants were engaged in premarital sex and having multiple sexual partners than non-migrants and their low utilization of condoms consistently emphasizes the need for promotion of condom use among these population groups.

Other findings observed in this study is the non-significant associations of the frequency of hometown visits, age, education, duration spent in migratory town and knowledge of HIV which are contrary to some studies reported earlier. HIV-related sexual behaviors among rural to urban migrants and non-migrants in China were independently associated with the duration of work in cities (34). In our study, this is probably due to social desirable responses in which participants might have failed to admit to have multiple sexual partners and unprotected sex because HIV-related sexual behaviors are not socially acceptable other tan this recall biases was a possibility as most of the responses were measured retrospectively.

These findings suggest that intervention programs should include increasing HIV-related knowledge, awareness of personal vulnerability to HIV infection and reduction of risk behaviors. To maximize the intervention effects, the program should focus on both rural to urban migrant as well as non-migrants. Given the high mobility of migrants, it is more practical to set up intervention programs targeting rural residents before they leave for towns for a temporary job and in the migratory towns.

Further research, particularly research that includes STIs and HIV testing, is needed to test rural to urban migrant and non-migrant differentials in prevalence of HIV and STIs. In addition the dynamic relationship between migration and HIV risk sexual behaviors need to be studied in detail to develop effective prevention program.

In summary the evidence availed in this study strongly suggest that, migration as a result of migrants detachment from family and home community should receive particular attention in intervention programs targeting temporary rural to urban migrants.

## CONCLUSIONS

Important socio-demographic determinants responsible for the risky sexual behaviors of both rural to urban migrants and non–migrants around Bure town and surrounding kebeles were identified which are crucial in designing HIV risk reduction interventions with potential policy implications. Education about safe sex and reducing numbers of sexual partners should be provided along with free condoms in rural areas (particularly those with a high incidence of urban migration) and among temporary rural to urban migrant communities in destination town. Furthermore, the local health care authority should consider offering low-cost primary health care and voluntary HIV counseling and testing for rural to urban migrants, which could greatly reduce their sexual risk taking and curb the bridging effect of migration in HIV transmission from urban to rural areas.

Some of the limitations observed in the current study are the study assessed only male rural to urban migrants and was restricted to the study woreda. Another possible limitation could be individual recall biases to questions concerning sexual behavior though such problems were minimized during the data collection process to respond to all the questions honestly as their responses would be helpful to improve the current national, local and workplace health policies. Although one might expect the limitations to affect generalization of the findings to the entire country, still the findings are relevant to rural residents and rural to urban male migrants, a rapidly growing urban workforce, in many Ethiopian towns or cities.
